# mRNA changes in nucleus accumbens related to methamphetamine addiction in mice

**DOI:** 10.1038/srep36993

**Published:** 2016-11-21

**Authors:** Li Zhu, Jiaqi Li, Nan Dong, Fanglin Guan, Yufeng Liu, Dongliang Ma, Eyleen L. K. Goh, Teng Chen

**Affiliations:** 1 College of Forensic Medicine, Xi’an Jiaotong University Health Science Center, Xi’an, Shaanxi, 710061, PR China; 2 The Key Laboratory of Health Ministry for Forensic Science, Xi’an Jiaotong University, Shaanxi, PR China; 3 Beijing Genomics Institute, Shenzhen, 518083, PR China; 4 Neuroscience Academic Clinical Programme, Duke-NUS Medical School, Singapore 169857; 5 Department of Research, National Neuroscience Institute, Singapore 308433; 6 Department of Physiology, Yong Loo Lin School of Medicine, National University of Singapore, Singapore 117597; 7 KK Research Center, KK Women’s and Children’s Hospital, Singapore 229899

## Abstract

Methamphetamine (METH) is a highly addictive psychostimulant that elicits aberrant changes in the expression of microRNAs (miRNAs) and long non-coding RNAs (lncRNAs) in the nucleus accumbens of mice, indicating a potential role of METH in post-transcriptional regulations. To decipher the potential consequences of these post-transcriptional regulations in response to METH, we performed strand-specific RNA sequencing (ssRNA-Seq) to identify alterations in mRNA expression and their alternative splicing in the nucleus accumbens of mice following exposure to METH. METH-mediated changes in mRNAs were analyzed and correlated with previously reported changes in non-coding RNAs (miRNAs and lncRNAs) to determine the potential functions of these mRNA changes observed here and how non-coding RNAs are involved. A total of 2171 mRNAs were differentially expressed in response to METH with functions involved in synaptic plasticity, mitochondrial energy metabolism and immune response. 309 and 589 of these mRNAs are potential targets of miRNAs and lncRNAs respectively. In addition, METH treatment decreases mRNA alternative splicing, and there are 818 METH-specific events not observed in saline-treated mice. Our results suggest that METH-mediated addiction could be attributed by changes in miRNAs and lncRNAs and consequently, changes in mRNA alternative splicing and expression. In conclusion, our study reported a methamphetamine-modified nucleus accumbens transcriptome and provided non-coding RNA-mRNA interaction networks possibly involved in METH addiction.

METH is an illegal psychostimulant that is highly addictive. Exposure to METH causes euphoria and hyperactivity, suggesting extensive changes in gene expression. A number of aberrantly regulated genes have been previously identified in various brain regions in response to METH. For example, METH exposure alters genes transcription involved in various biological functions in the striatum, including GTPase signaling, apoptosis, and cell cycle control^1^,^2^,^3^,^4^. Recently, an extensive mRNA and miRNA analysis in the ventral tegmental area (VTA) of rats following METH self-administration procedure showed differentially expressed mRNA and miRNA^5^. Thus, it is of importance to study gene expression changes and the regulation of these changes, specifically in the brain regions related to drug addiction.

Post-transcriptional gene regulation involving non-coding RNAs (ncRNAs), such as miRNAs and lncRNAs, are known to play a critical role in drug addiction. Previous findings demonstrated miRNAs as significant modulators in controlling the impacts of abused drugs such as cocaine, nicotine, morphine and opioids, on brain rewarding circuits and the development of addiction-related behaviors^6^,^7^,^8^,^9^,^10^. LncRNAs are known to control genes expression through various mechanisms and are likely to influence brain development, neuronal plasticity, and neural disease^11^,^12^,^13^,^14^,^15^. Many lncRNAs were found induced by cocaine and heroin in the nucleus accumbens (NAc)^12^,^16^, suggesting a potential role of lncRNAs in mediating drug addiction. We recently analysed METH-induced ncRNAs changes in the NAc of mice by using RNA sequencing technologies^17^,^18^ and identified numerous METH-responsive miRNAs and lncRNAs. However, the potential post-transcriptional regulations of those ncRNAs on METH-induced mRNA changes have not been studied. Changes in expression of mRNA, specifically various mRNAs isoforms differentially spliced from a single gene can also be regulated post-transcriptionally by alternative splicing. Evidences both from in *vitro* non-nervous tissues and *in vivo* cocaine-treated mouse model suggested a regulatory role of alternative splicing for specific genes^19^,^20^. These changes in alternative splicing in response to cocaine expanded and diversified the proteome modulating drug addiction-related changes^21^. However, the contribution of alternative splicing to METH action is still unclear.

Although gene expression changes and their post-transcriptional regulation are implicated to play important roles in drug addiction, there is still insufficient data for METH-mediated changes in the NAc. Therefore, we sought to study genome-wide METH-induced mRNA expression in the NAc using ssRNA-Seq. The post-transcriptional regulation of mRNA by miRNAs, lncRNAs and alternative splicing were analyzed to reveal the potential regulatory consequences in response to METH.

## Results

### Overview of the ssRNA-Seq data

**Figure 1:**
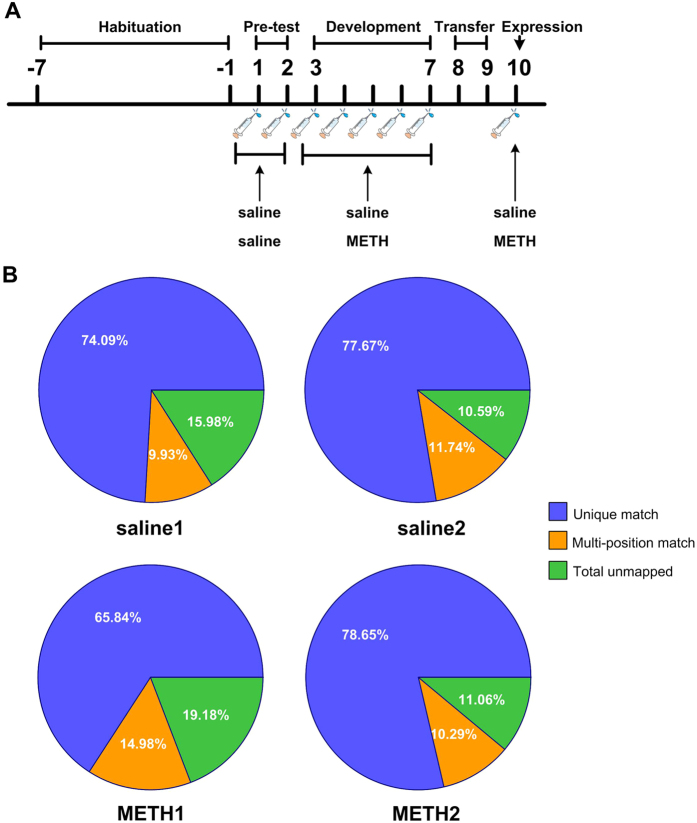
Treatment schedules and overview of the ssRNA-Seq data. (**A**) The schematic timeline of METH treatment regimen. Habituation (day (−7)–(−1)): The mice were handled daily for 1 week to adapt to the experimenter before treatment. Pre-test (day 1-2): mice were given once daily injections of saline for two consecutive days. Development (day 3–7): After pre-test, mice were divided into two groups randomly, and were given once daily injections of METH (2 mg/kg) or saline for five consecutive days. Transfer (day 8–9): two groups of mice were experienced two injection-free days. Expression (day 10): mice were given a challenge injection of either 2 mg/kg dose of METH or saline. Horizontal locomotor activity was performed 60 minutes before and after the injections on all drug treatment days. (**B**) Statistical alignment of the sequencing data from the saline and METH groups. Unique mach represents the mapped reads that aligned to only one position in the mouse genome. Multi-position mach represents the mapped reads that aligned to more than one positions in the mouse genome. Total unmapped indicates reads that did not match to the mouse genome.

**Figure 2:**
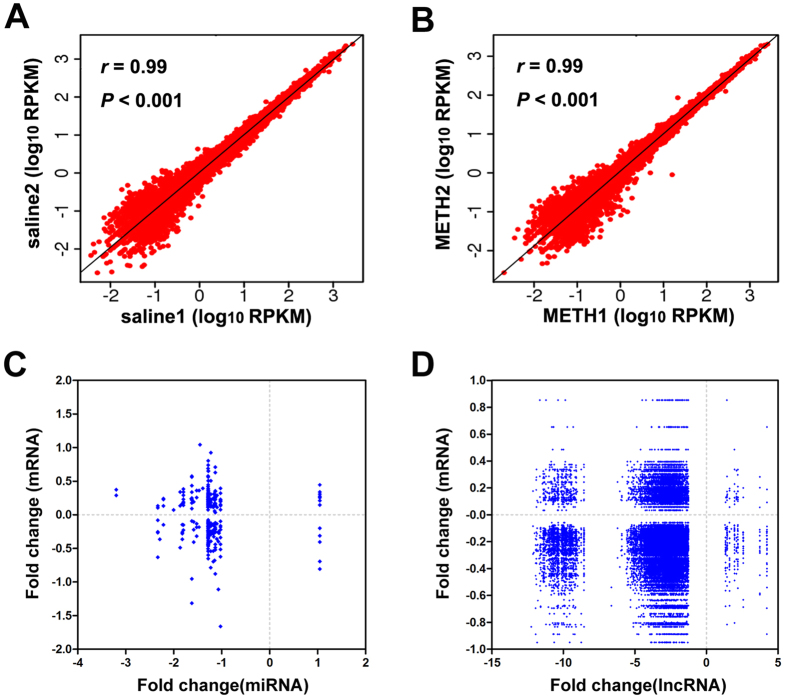
Expression profiles of mRNAs within each group and correlated interactions formed by METH-induced ncRNAs-mRNAs. Pearson correlation scatter plot comparing log_10_RPKM of shared pcGenes that expressed both in saline1 and saline2 samples (**A**, r = 0.99, *P* < 0.001) and in METH1 and METH2 samples (**B**, r = 0.99, *P* < 0.001) showed nearly equivalent expression profile within the samples of each group. RPKM: The number of uniquely mapped reads per kilobase per million mappable reads. Interaction networks formed by miRNA-mRNAs and lncRNAs-mRNAs were shown by the fold change of miRNAs (**C**, x-axis) and lncRNAs (**D**, x-axis) identified in NAc of mice following METH exposure^17^,^18^ and their potential target genes with significant changes (y-axis) in response to METH (current study).

To examine transcriptional changes in response to METH, we used a METH administration regimen that has been previously demonstrated to produce robust locomotor sensitization. Animals were randomly allocated to the METH (METH1 and METH2) or saline-treated (saline 1 and saline 2) groups. Mice were injected daily with 2 mg/kg of METH or saline through the intraperitoneal route for 5 consecutive days followed by two injection-free days and a final challenge injection (Fig. 1A). These mice were sacrificed 24 h after the final injection and samples prepared for ssRNA-Seq to examine for transcriptome changes in the NAc of mice. An average of 50.5 million clean reads per sample passed filtering and we observed no difference in the alignment reads between groups (Fig. 1B). Assembling and annotation of the mapped reads have shown that an average of 15497 and 15396 mRNAs were expressed (reads > 1.0) in both samples from the saline and METH groups respectively. Given the nearly equivalent values for each mRNA within the samples of each group (Fig. 2A,B), we used average reads of the shared mRNAs from each group to represent their expression levels in saline and METH subjects in the following analyses.

### METH treatment induced aberrant expression of mRNAs in the NAc of mice

Under a combination of statistical significance (*P* < 0.0001, FDR ≤ 0.00001), the differential expression analyses showed 2171 mRNAs exhibited differentially expressed level in the NAc of METH-treated mice, including 1059 up-regulated and 1112 down-regulated mRNAs (full list shown in Supplementary Table S1). Changes in mRNA expression identified differentially expressed genes (DEGs) and several of these DEGs have been implicated in the regulation of neuroplasticity and drug addiction, such as *Arc* (2.06-fold), *Junb* (1.68-fold), *Fos* (1.89-fold) and *Ntrk1* (1.61-fold) (Supplementary Table S1).

In order to reveal the molecular and cellular functional characteristics affected by METH exposure, we subjected all of the DEGs to Gene Ontology (GO) analysis. As shown in Table 1, we observed significant effects of METH exposure on protein-coding genes (pcGenes) involved in synaptic transmission, synaptic plasticity, mitochondrial inner membrane functions, and oxidative phosphorylation. In addition, METH treatment also altered the expression of pcGenes that are responsible for viral infectious cycle.

### Non-coding RNA-mRNA networks revealed biological functions in response to METH

METH have been shown to regulate expression levels of miRNAs and lncRNAs in the NAc of mice^17^,^18^, suggesting potential gene regulatory consequences following METH exposure. To investigate the post-transcriptional regulatory relationships between DEGs and ncRNAs, we performed regulatory analysis by aligning these DEGs to the putative targets for METH-responsive miRNAs and lncRNAs that we previously identified^17^,^18^. We found 309 DEGs (168 up-regulated and 141 down-regulated) that are putative targets for 32 METH-responsive miRNAs (31 down-regulated and one up-regulated, Table 2). These miRNAs-DEGs networks exhibited both positively and negatively correlated interactions (Fig. 2C). Positively correlated interactions refer to down-regulated miRNA and down-regulated DEGs, while negatively correlated interactions refer to down-regulated miRNA and up-regulated DEGs. Examples of negatively correlated interactions include miR-212-3p, miR-338-3p and miR-138-5p and their potential targets involved in neural functions, *Arc, Fos* and *Ntrk1* respectively. These neural function-related DEGs were upregulated while their interacting miRNA were downregulated upon METH treatment^17^. We also found 586 DEGs (109 up-regulated genes and 477 down-regulated genes) associated with genes for 3988 differentially expressed lncRNAs in response to METH. The lncRNAs-DEGs networks also exhibited both negatively and positively correlated interactions (Fig. 2D).

**Figure 3:**
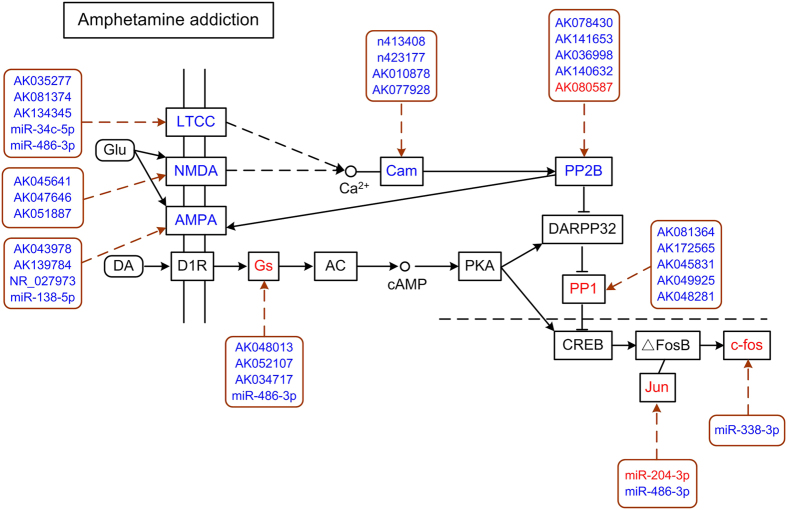
Predicted signaling pathways regulated by differentially expressed miRNAs and lncRNAs in response to METH. Pathway analysis of DEGs that were putative target genes of differentially expressed miRNA and lncRNAs was carried out to assess their putative biological functions (Table 2). As examples, amphetamine addiction pathway composed of DEGs and their corresponding miRNAs and lncRNAs was shown. Genes with red font suggested an up-regulation while genes with blue font indicated a down-regulation in response to METH.

We next carried out pathway analysis for those DEGs that are associated with changes in miRNA or lncRNA to find potential functions of these associations. This pathway analysis was done using Kyoto Encyclopedia of Genes and Genomes (KEGG, http://www.kegg.jp/), a bioinformatics tool based on external pathway data sources. All significantly enriched pathways (*P* < 0.05and EF > 2.0) were shown in Table 3. Expectedly, both miRNA targets and lncRNA associated genes showed significant enrichment in pathways that were verified to be responsible for drug addiction, such as amphetamine addiction, glutamatergic synapse, dopaminergic synapse, and MAPK signalling pathway (Table 3). Presented here are pathways of amphetamine addiction (Fig. 3) and glutamatergic synapse (Supplementary Fig. S1), regulated by differentially expressed miRNAs and lncRNAs associated with changes in DEGs in the NAc of mice following METH treatment. Several significantly altered DEGs involved in vibrio cholerae infection and mTOR signalling pathway were also predicted to be regulated by both METH-induced changes in miRNAs and lncRNAs (Table 3). Pathways regulated by METH-dependent miRNAs-DEGs networks include metabolic pathways, such as fatty acid elongation, arginine and proline metabolism, oxidative phosphorylation, biosynthesis of secondary metabolites. Several other signalling pathways such as ErbB signaling pathway, phosphatidylinositol signaling system and wnt signaling pathway were predicted to be modulated by METH-dependent lncRNAs-DEGs networks. Interestingly, we also observed DEGs that function in ribosome, spliceosome and protein export are potentially modulated by miRNAs, suggesting an alternative splicing regulation following METH exposure.

### METH-induced variation on alternative splicing

**Figure 4:**
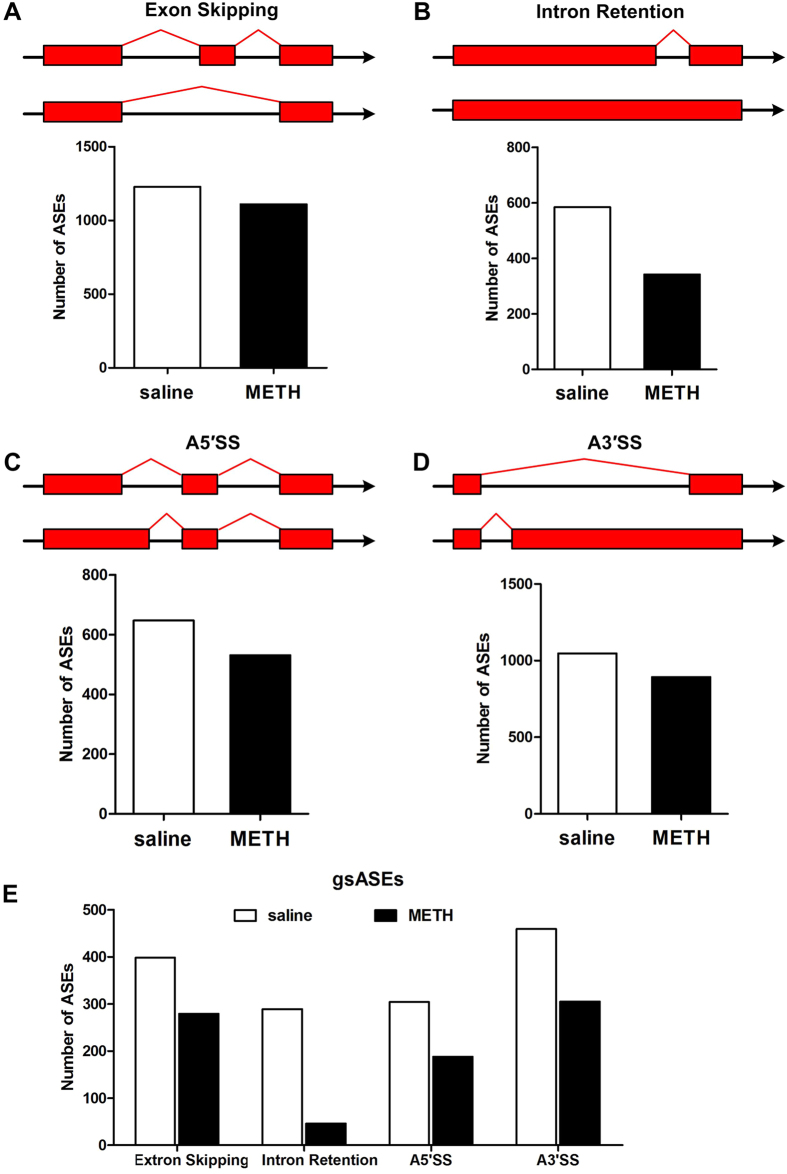
Variations on alternative splicing for genes transcription. The ASEs types including exon skipping, intron retention, and alternative 5′and3′splicing sites (A5′SS and A3′SS) were analysed. The number of evens showing exon skipping (**A**), intron retention (**B**), A5′SS (**C**) and A3′SS (**D**) in saline and METH groups of mice were shown respectively. (**E**) Number of group-specific alternative splicing events. The number of genes corresponding to group-specific alternative splicing events was shown in y-axis.

To determine the splicing differences in response to METH, we analyzed alternative splicing events (ASEs) on all clean data of each group. The ASEs analysis included exon skipping, intron retention and alternative 3′and 5′splicing sites (A3′SS and A5′SS). The numbers of ASEs corresponding to each type in both saline and METH-treated groups were identified and calculated. As observed previously, the most frequent event type in both groups was exon skipping, followed by A3′SS, A5′SS and intron retention^22^. However, the numbers of ASEs in METH-treated group were about 10–40% less than those in saline-treated group (Fig. 4A–D). As compared to all other ASEs, the intron retention events in METH-treated group showed the biggest decrease of about 40%. Further analysis considering group-specific alternative splicing events (gsASEs), defined as a splicing event identified only in one group but not in the other group, showed a total of 1450 events that were saline-specific splicing variants while a total of 818 events were METH-specific (Fig. 4E).

### Validation of changes in gene expression using qPCR

**Figure 5:**
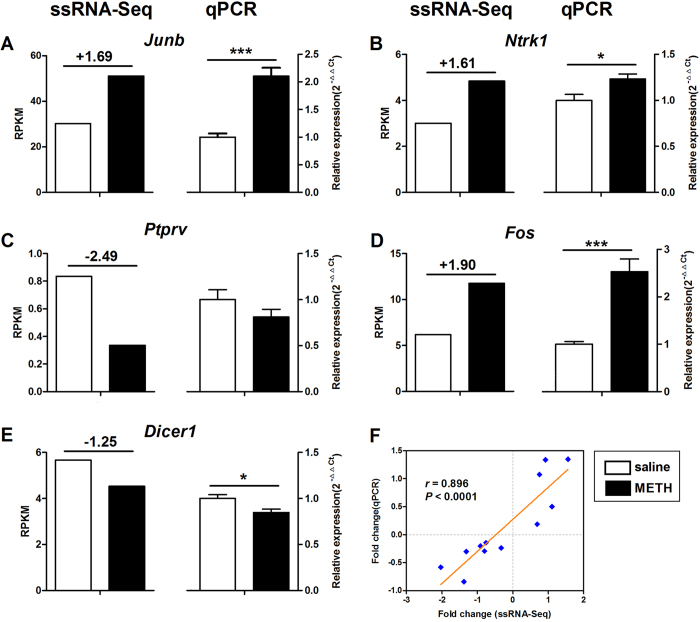
Confirmatory qPCR of DEGs selected from ssRNA-Seq analysis. Left panels exhibited changes in RPKM detected by ssRNA-Seq. The number above the bars indicated fold change. Right panel depicted changes identified through qPCR. The expression levels were calculated relative to *Gapdh*. Values are presented as the means ± SEM. Independent-samples *t*-test, *P < 0.05.Pearsoncorrelation scatter plot comparing the fold change for all selected genes in the NAc as analysed from ssRNA-Seq data (x-axis) and qPCR data (y-axis) was shown in F. R represents the Pearson’s coefficient.

To validate changes in gene expression from the ssRNA-Seq analysis, confirmatory qPCR was conducted on 12 DEGs. Genes were selected based on fold change, functional enrichment, and potential role in drug addiction. Seven of these genes (*Junb, Ntrk1 Fos, Dicer1, Rps23, Avpr1a* and *Gbp4*) were significantly changed in METH-treated mice, consistent with the ssRNA-Seq data. The other five genes examined (*Ptprv, Mrpl52, Rgs11, Rreb1, Ligp1*) also showed increasing or decreasing trend, that are consistent with our ssRNA-Seq data. Thus, these data verified the consistency between the two independent techniques used for the study. The differential expression of genes known to be involved in drug addiction (*Junb, Ntrk1, Ptprv, Fos*) and miRNA regulation (*Dicer1*) are presented in Fig. 5A–E with their paired ssRNA-Seq results. The other seven genes are presented in the Supplementary Figure S2. The comparison of the fold change obtained using qPCR and ssRNA-Seq indicated a strong correlation between both techniques (Fig 5F, *r* = 0.896, *P* < 0.0001). In summary, these confirmations by qPCR indicated a strong reproducibility of changes in genes expression by using another independent technique.

## Discussion

High-throughput genome-wide approaches have been widely utilized in animal models to provide an overview of global gene regulation pattern, specifically in relation to various diseases. In this study using ssRNA-Seq, we identified 2171 DEGs in the NAc of METH-treated mice. Many of these DEGs are known to be critical for brain functions. GO analysis showed significant enrichment of METH-responsive genes involved in neuronal plasticity, mitochondrial energy metabolism and immune response, based on GO-defined changes in molecular and cellular functions and biological processes. Previous studies demonstrated that exposure to abused drugs results in neural dysfunction that is critical for modulating addictive behaviors^23^,^24^,^25^. Thus, it is not surprising that GO analysis show relatively higher number of genes involved in neural functions.

Given that METH is a powerful psychostimulant that can produce robust rewarding effects through modulating the dopaminergic synapse, we expectedly found several DEGs (such as *Comt, Akt, Gsk3a/b, PP2A, Slc family* and *PLC*) that are presented in the dopaminergic synapse pathway (KEGG pathway database). These genes were either related to dopamine re-uptake (Comt, *Slc* family) or dopamine- regulated downstream signals (*Akt, Gsk3a/b, PP2A*, and PLC). Interestingly, we found some overlaps in METH- and cocaine-responsive genes. 136 DEGs in NAc of METH-treated mice were also changed in the NAc of mice treated with cocaine (Supplementary Figure S3)^12^. Although cocaine can also produce rewarding effects through interaction with the mesolimbic dopaminergic system, we did not find any dopamine-related changes among these overlaps. Therefore, the observed gene changes from the treatment with these two different drugs suggest the presence of common and differential mechanisms in mediating METH and cocaine addiction. Further studies are needed to investigate these common and differential pathways and mechanisms in drug addiction.

METH treatment also altered the expression of pcGenes that are involved in other non-neural functions. Notably, we found a large number of mitochondrial energy metabolism changes in response to METH, suggesting a neurotoxic response may have been induced in the brain by the METH regimen used in the current study. This result is consistent with the previous studies. Based on extant literatures, either a single administration or repeated injections of METH could causes energetic metabolism impairment in the amygdala, prefrontal cortex, hippocampus and striatum of rats accompanied with a significant behavioral sensitization^26^,^27^. Mitochondrial energy metabolism provides energy source responsible for brain functions and mitochondrial energy metabolism disruptions are known to play important roles in psychiatric disorders such as schizophrenia, bipolar disorder, and major depression disorders^28^,^29^. Thus, the effects of METH exposure on mitochondrial functions suggest that the molecular adaptations of cells responsible for a large extent of brain energy metabolism may play a role in METH addiction. In addition, we found significant changes in ionotropic glutamate receptor genes, such as *Grin2a, Grin2b* and *Grin2d*. Considering that mitochondria in nerve terminals are capable of maintaining cellular calcium (Ca^2+^) homeostasis^30^,^31^ and neuronal mitochondrial Ca^2+^ processing supports Ca^2+^ influx via ionotropic glutamate receptors^32^, these ionotropic glutamate receptor alterations may possibly involved in the dysregulation of mitochondrial calcium load contributing to METH addiction.

Exposure to METH also causes inflammatory response that may play a role in METH-induced neuronal injury^33^. Furthermore, there is increasing evidence showing METH (both at low and high doses) induces a heightened inflammatory environment in the brain, that affects the regulation of cellular immune responses^34^,^35^. In line with these literatures, we found that METH treatment altered the expression of pcGenes that are involved in immune response. Therefore, how METH having divergent impacts on other responses in addition to neural functions warrants further investigations.

In addition to the mRNA expression patterns, the underlying molecular regulatory networks are also important to understand how various mRNA changes in response to METH can regulate potential functions. Previously, we have identified a subset of METH-responsive miRNAs and lncRNAs in the NAc of mice and predicted their target genes using bioinformatics tools. Thus, in order to determine how non-coding RNAs are involved in METH-induced various mRNA changes, we performed a comparison between the DEGs and the potential target genes of miRNAs and lncRNAs. We identified numerous potential METH-responsive miRNA-mRNA and lncRNA-mRNA networks, which suggested changes in the complex gene regulatory networks upon METH exposure. Since miRNA is known to suppress mRNA expression and considering the large down-regulation pattern of miRNAs^17^, our data revealed a larger negatively correlated networks (down-regulated miRNAs with up-regulated mRNAs) and a smaller positively correlated networks (down-regulated miRNAs with down-regulated mRNAs). This suggested that the up-regulation of these relevant mRNAs maybe due to the down-regulated miRNAs in response to METH. The transcription data also revealed coordinative changes between mRNAs and miRNAs while the putative target genes of METH-regulated miRNAs did not show global changes. These data indicated that the effects of METH exposure on genes transcription were not only regulated by miRNAs but may also be resulting from direct effects of METH or indirect effects of other regulators.

Besides miRNA-mRNA networks, we also identified complex interaction networks formed by lncRNAs-mRNAs. LncRNAs are demonstrated to be transcription and epigenetic modulators, and are involved in various mechanisms to regulate genes expression. Unlike miRNAs, lncRNAs are able to increase or decrease the expression of pcGenes. Thus, we observed a more evenly distribution of both negatively and positively correlated interactions with mRNAs. This study identifying potential METH-responsive lncRNA-mRNA interaction networks, suggests a novel regulatory role of lncRNAs following METH exposure.

The altered ncRNAs-mRNAs networks underscored the biological importance of induction of ncRNAs targeting in response to transcriptional regulation induced by METH. Therefore, we performed KEGG pathway analysis on METH-responsive gene lists for miRNAs and lncRNAs, and observed a significant enrichment of miRNA-mRNA and lncRNA-mRNA on pathways involved in drug addiction (Table 3). For example, predicted METH-related targets include a range of amphetamine addiction- and synaptic plasticity-related receptors and downstream signals, such as glutamatergic receptors (*Grin2a, Grin2b, Gria1, Gria3, Gria4*), *Gnas, Jun* and *Fos* (Fig. 3 and Supplementary Figure S1). In addition, we also identified that up-regulated genes implicated in the regulation of neuroplasticity such as *Arc* and *Ntrk1*^36^,^37^ were potential targets for miR-212-3p and miR-138-5p respectively. Both miR-212-3p and miR-138-5p were significantly down-regulated in response to METH and were previously demonstrated as important regulators of cocaine addiction and neuronal plasticity^7^,^38^. These results suggested that ncRNAs are involved in the modulation of METH addiction. Moreover, we also observed enrichment of miRNA-mRNA and lncRNA-mRNA on immune response related pathways, such as vibrio cholerae infection, suggesting an involvement of ncRNAs in the METH-induced immune dysfunction. These results indicated the effects of METH on neural and other non-neural systems were, at least partially, dependent on changes of miRNAs and lncRNAs.

Alternative splicing is known as another kind of the post-transcriptional mechanisms for generating diverse gene products. Abnormal changes in splicing can result in disease consequences^39^. Interestingly, we found that *Dicer1*, an endonuclease responsible for the generation of miRNAs from their precursors^40^, was significantly down-regulated in response to METH. This finding implicated dysregulation of splicing of miRNAs in the NAc of METH-treated mice^17^. We also identified contribution of changes in alternative splicing induced in NAc of METH-treated mice (Fig. 4). Although there are a few recent studies on splicing regulation of genes transcription in addiction models^20^,^41^,^42^, our data is the first comprehensive analysis of METH-induced splicing regulation in NAc of mice. These data underscored the importance of discrimination of isoform differences on total gene transcription and also expanded the degree to which METH modified the transcriptome in NAc. In addition, the impact of spliceosome on pre-mRNA splicing requires interactions of pre-mRNA with splicing factors. Our previous work revealed that lncRNAs such as *Neat1, Neat2* and *Miat*, function as cofactors for pre-mRNA splicing through interaction with splicing factors^43^,^44^,^45^, were significantly down-regulated in response to METH. Moreover, we identified that miRNAs might modulate DEGs that function in ribosome and spliceosome, critical for pre-mRNA splicing. These results indicated that alternative splicing regulation of genes transcription in drug addiction may require ncRNAs-related nuclear modification.

Several limitations should be considered in the present study using bioinformatics tools to relate our DEG data to the ncRNA data. It is important to note that changes in non-coding RNA and mRNA do not always directly translate to the expression and functions of proteins. Although we currently observed a large number of mRNAs variants, but variations of protein products and changes of the localization and ultimate function of these variant protein products requires more intensive investigations. Subsequent verifications are then required to determine if manipulating the expression of the ncRNAs can recapitulate the effects of METH and/or alter METH-induced DEGs. Another limitation is the currently available methods and technologies to efficiently isolate pure core and shell region as well as different neuron subpopulations within each region. The core and shell subregions of the NAc are components of distinct microcircuits and exhibit different drug-induced changes^46^. However, the core and shell regions of the NAc are not distinguished in the present study. Nonetheless, this is the first description of the global mRNAs expression profiling and overview of post-transcriptional regulation in the NAc by miRNAs and lncRNAs in the context of METH-induced behavioral sensitization. Future studies include identifying the precise regulatory mechanisms of each of these ncRNAs-DEGs networks, and also the distinct changes and functions between the different neuron subpopulations in different regions of the NAc. This may further strengthen our understanding of the relationships between the ncRNAs-DEGs networks and METH addiction.

In summary, we reported a METH-modified transcriptome in the NAc and provided an overview of post-transcriptional regulation by miRNAs, lncRNAs and alternative splicing upon METH exposure. Functional analysis of all DEGs and the DEGs correlated with changes in miRNAs and lncRNAs revealed that the effects of METH on neural and other different systems were, at least in part, dependent on changes of miRNAs and lncRNAs. A large number of METH-mediated changes in mRNA variants and the identification of alternative splicing changes expanded the current view on how METH can modify the transcriptome. Our results provided important information concerning the basic mechanisms and potential consequences of post-transcriptional regulation in response to METH *in vivo*.

## Methods

### Animals

Adult wild-type C57BL/6 mice (7-8 weeks old, male, 20–25 g), purchased from Beijing Vital River Laboratory Animal Technology Co. Ltd, were housed in cages (4 per cage) within regulated environment (23 ± 1 °C, 50 ± 5% humidity) with 12-h light/dark cycle (lights on from 7:00 am to 7:00 pm). The mice had ad libitum access to food and water and were handled daily for 1 week for adaptation to the experimenter before treatment. The experiments involving animals were approved and all treatment procedures were carried out in accordance with guidelines by the Institutional Animal Care and Use Committee of Xi’an Jiaotong University.

### METH treatment and NAc dissection

METH hydrochloride used in this study was purchased from the National Institute for the Control of Pharmaceutical and Biological Products (Beijing, PR China) and was dissolved in sterile physiological saline. The METH injection paradigm used in this study has been demonstrated in previous studies to produce robust locomotor sensitization^47^,^48^. Mice were given once-daily injections of saline for two consecutive days (day 1-2), after which they were randomly grouped into two. The groups of mice were then received once-daily intraperitoneal (i.p) injections of METH (METH group, 2 mg/kg/injection, n = 16) dissolved in sterile physiological saline or saline (saline group, n = 16) for five consecutive days (day 3–7). After five injected days, the mice were housed in cages for two injection-free days (day 8–9). On day 10, the mice were received a challenge injection of either METH or saline. Before the beginning of the experiments, the mice were brought into the experiment room for 1 h to adapt to the experimental environment. Meanwhile, on all drug treatment days, horizontal locomotor activity was performed in the open field apparatus (43 cm × 43 cm × 43 cm) 1 h before and after the injections. Previous studies including our own works have shown changes in gene expression at 24 hr in drug-induced models to allow for evaluation of the steady state response^12^,^17^,^18^,^49^,^50^. For comparison and consistency, the mice used in the present study were also sacrificed 24 hr after the final injection. The brains were removed quickly and the NAc (+1.70 mm from Bregma) harvested according to the brain atlas of Paxinos and Franklin^51^ were immediately frozen in liquid nitrogen until use. Sixteen mice per group were used to generate two independent samples of eight mice each and NAc lysates of eight mice from each group were pooled as one sample for total RNA isolation.

### RNA extraction

Total RNA was extracted using TRIzol following instructions of the manufacturer (Invitrogen, USA). Given two independent samples were prepared for each group, the RNA samples of each group were referred as saline1, saline2 and METH1, METH2. Samples were eluted in RNase-free H_2_O, and quantified using Agilent 2100 BioAnalyzer (Agilent Technologies, USA). All samples were sufficient to construct the strand-specific cDNA libraries (>180 ng/μl total RNA) and a RIN > 8.0 were accepted for RNA sequencing analysis.

### ssRNA-Seq

ssRNA-Seq were carried out by the Beijing Genomics Institute Shenzhen (BGI Shenzhen, China). Total RNA (5 μg) from each sample was used for construction of strand-specific cDNA libraries, which were prepared as previously described^18^. The cDNA libraries were evaluated using the Agilent 2100 BioAnalyzer and then sequenced on an Illumina HiSeq 2000. The sequencing reads alignment and assembling were done using TopHat v2.0.4^52^ and Cufflinks v2.0.0^53^ packages after filtering off dirty reads. The mRNA transcripts were annotated by perfect sequence mapping to the databases, including KEGG Orthology, non-redundant protein database(ftp://ftp.ncbi.nih.gov/blast/db/FASTA/),COG(http://www.ncbi.nlm.nih.gov/COG/) and UniProtKB/Swiss-Prot^54^. For relative expression analysis, the number of uniquely mapped reads per kilobase per million mappable reads (RPKM) for each pcGene was calculated^55^. RPKM across samples of each group were then averaged because the values for each transcript were very consistent. The fold change in expression between METH- and saline-treated mice was obtained by dividing RPKM_METH_ by RPKM_saline_. The alternative splicing analyses was performed with SOAPsplice^56^ and the alternative splicing events of each sample were counted in each group. Only those events appeared in both samples of each group were included for further analysis.

### Functional enrichment

The differentially expressed miRNAs and lncRNAs data used for all comparison analysis in this study were previously identified by our published works^17^,^18^. The potential target genes of these miRNAs and lncRNAs were mapped to the differentially regulated pcGenes identified here. For functional annotation, we used Blast2GO^57^ to analyze the GO and KEGG pathway enrichment for the DEGs and calculated the enrichment factor (EF) for each term or pathway. The *P* values were calculated via hypergeometric tests and go with a correction. Only those GO terms or KEGG pathways with enrichment of corrected *P* < 0.05 and EF > 2.0 were included.

### qPCR assay

cDNAs were synthesized from total RNA of the NAc of individual mice using Thermo Scientific RevertAid First Strand cDNA Synthesis Kit (Thermo Scientific, USA). Based on the manufacturer suggested parameters (25 °C 5 min, 42 °C 60 min, and 70 °C 5 min), 500 ng of total RNA from each sample was utilized for each reaction. qPCR was performed on Bio-Rad iQ5 system (Bio-Rad, USA) using SYBR Premix Ex Taq II (TaKaRa Biotechnology, Japan) under the following conditions: 95 °C for 30 sec; 95 °C for 10 sec and 60 °C for 1 min, repeated for 40 cycles. *Gapdh* was used as an endogenous control for the qPCR, and the relative expression levels were determined by the 2^−△△Ct^ method^58^. Primer pairs were chosen to keep the melt temperature (Tm) between 56 °C and 62 °C and products in the100–200bp size range (Supplementary Table S2).

### Statistical analyses

The variances in sequencing assays were directly estimated by the Poisson distribution^59^ and a false discovery rate (FDR)^60^ for each pcGene was calculated to compensate for false-positive findings at each significance threshold. The following criteria: *P* < 0.0001, FDR ≤ 0.00001 was used to set up differentially regulated pcGenes. For the significance of qPCR assay, we performed an independent-sample t-test and data with significance *P* < 0.05 were considered to be differentially expressed (SPSS v17.0, SPSS Inc., USA). Pearson’s coefficient analysis was also performed with SPSS (version 17.0, USA).

## Additional Information

**How to cite this article**: Zhu, L. *et al*. mRNA changes in nucleus accumbens related to methamphetamine addiction in mice. *Sci. Rep.* **6**, 36993; doi: 10.1038/srep36993 (2016).

**Publisher's note:** Springer Nature remains neutral with regard to jurisdictional claims in published maps and institutional affiliations.

## Supplementary Material

Supplementary Information

Supplementary Dataset 1

## Figures and Tables

**Table 1:** GO enrichment of DEGs.

GO term	EF^1^	*P* –value^2^
Cellular component		
GO:0015934 large ribosomal subunit	3.42	3.50E-07
GO:0033267 axon part	3.00	1.21E-07
GO:0005840 ribosome	2.92	2.18E-11
GO:0005743 mitochondrial inner membrane	2.86	3.38E-07
GO:0030425 dendrite	2.86	1.25E-07
GO:0030427 site of polarized growth	2.84	1.22E-06
GO:0030136 clathrin-coated vesicle	2.66	2.18E-08
GO:0045202 synapse	2.65	2.40E-06
Molecular function		
GO:0005198 structural molecule activity	2.19	2.25E-11
GO:0019900 kinase binding	2.00	1.30E-05
Biological process		
GO:0019058 viral infectious cycle	3.79	1.48E-16
GO:0001505 regulation of neurotransmitter levels	3.51	4.54E-11
GO:0048167 regulation of synaptic plasticity	3.51	1.53E-06
GO:0022415 viral reproductive process	3.43	3.50E-16
GO:0007268 synaptic transmission	3.04	3.33E-14
GO:0050804 regulation of synaptic transmission	2.85	1.36E-10
GO:0023061 signal release	2.77	9.87E-09
GO:0003001 generation of a signal involved in cell-cell signaling	2.77	9.87E-09
GO:0051969 regulation of transmission of nerve impulse	2.63	1.76E-10
GO:0006887 exocytosis	2.63	1.80E-06
GO:0031644 regulation of neurological system process	2.57	1.06E-10
GO:0015980 energy derivation by oxidation of organic compounds	2.52	2.73E-08
GO:0048667 cell morphogenesis involved in neuron differentiation	2.44	3.57E-06
GO:0006091 generation of precursor metabolites and energy	2.35	9.69E-10
GO:0055114 oxidation-reduction process	2.28	1.46E-08
GO:0007610 behavior	2.12	3.90E-10
GO:0006412 translation	2.06	3.67E-08

^1^indicated enrichment factor.

^2^*P* values were calculated via hypergeometric tests and go with a correction.

**Table 2:** Full list of miRNAs-DEGs.

miRNA	Fold change	Up-regulated genes	Down-regulated genes
miR-101a-3p	−1.177	*Spire1*	*Ccnt2*
miR-101b-3p	−1.096	*Txndc17,Spire1*	*/*
miR-106b-5p	−1.104	*/*	*St3gal5,Mrpl21*
miR-127-5p	−1.798	*Arxes2,Arxes1,Txndc17, Cntfr,Cpe,Gpx4,Ttyh3*	*Stmn4,Tbc1d24,*
miR-138-5p	−1.285	*Ntrk1,Nrarp,Trib1,Cachd1, Eif3f, Srm,Plxnb1,Cntfr,Slc1a4, Fam195b,Rad21,Gnb2l1, Atp6ap1,Nrgn*	*Ncor1,Gria4,Ubtd2,Ccnl2, Igf2*
miR-145a-5p	−1.175	*Atp6v0b,Usp11*	*Agtpbp1,Nrcam,Gripap1,Son, Col11a1*
miR-153-3p	−2.289	*/*	*Unc13b*
miR-204-3p	1.044	*B3gat2,Prr7,Atp1b2,Jun,Aqr, Tmem179,Rpl31*	*Mylk,Zfp788,Nrn1,Col11a1, Caprin2*
miR-212-3p	−1.452	*Arc*	*/*
miR-218-5p	−1.165	*/*	*Rabgap1l*
miR-299a-3p	−1.056	*/*	*Dnmt3a*
miR-29a-5p	−1.584	*Use1*	*Rps24,Akap5,Chd7*
miR-29b-3p	−2.214	*Prpsap1*	*/*
miR-29c-3p	−1.634	*/*	*Hba-a1*
miR-300-3p	−1.228	*Pigs,Wars*	*Eif2s3x,Lmo3,Mcam,Rhobtb1*
miR-301a-5p	−1.165	*Guk1*	*Arglu1*
miR-3068-5p	−1.522	*Rps7,0610031J06Rik*	*Snn,Rabgap1,Slco1a4*
miR-30e-5p	−2.002	*Tac1*	*/*
miR-324-5p	−1.133	*Acot1,Agt,Pygm,Pcdh8,Por, 2310022A10Rik,B4galt3, Ergic3,Hgs,Mllt1,Ranbp3, Htra1, Glul*	*Nov,Tulp4,Tlk1,Znf512b, Klhl23, Ankrd33b,Camk4, Cdc42bpa,Ccnl2,Inha, Neurod6, Gbp6*
miR-33-5p	−3.198	*Pdyn,Aurkaip1*	*/*
miR-338-3p	−1.226	*Fos,Ramp3,Ndufb10,Wrnip1, Slc1a4,Nckipsd,Kctd17, Arl6ip5,Psmd3,Abhd8*	*Gucy1a3,Serpini1,Ndst1, Tmod2,Lrfn5,Ndufa3,Acsl4, Tmed9*
miR-341-3p	−2.215	*Nme2,Cops7a*	*Hipk3*
miR-345-5p	−1.149	*Slc7a10,Mlc1,Evl2,Pomp, Tsc22d1,Hspa52,Pde2a*	*Brwd1,Brwd1,Nktr*
miR-34c-5p	−1.026	*Rnd2,Pycrl,Rpl11,Comt,Ptpn1, Ctbp1,Fxr2,Ppp2r5b,Zmiz2*	*Pde1b,Tomm20,Negr1,Mapk8,Dlgap1,Cacna1d,Slc13a4, Gprin3,Nsun7,Gm20594*
miR-378c	−1.822	*Ngef*	*Napb,Myt1l,Zranb2,Rars2*
miR-434-5p	−1.394	*Mxd4*	*/*
miR-503-5p	−1.622	*Ngfr,Egr4,Grin2d,Ift27,Pex6, Ttll1,Pnck,Cacng3,Cxcl14,Lrp1*	*Thy1,Srp54b,Ptprv*
miR-551b-3p	−2.332	*Cdk5r2,Ntrk2,Rrp1*	*Spag9,Dyrk1a,Rsf1,Slc16a7*
miR-708-3p	−1.471	*Dlx2*	*St8sia3*
miR-873a-5p	−1.867	*Chchd6,Tmem66*	*Acta2*
miR-99b-3p	−1.070	*/*	*Gtpbp2,Vtn,Rad50,Gbp5*
miR-486-3p	−1.282	*Npas4,Ndufab1,Zfp36,Btg2, Egr4,Tmem107,Ramp3, B3gat2,Ecel1,Dusp6,Dbndd1, Aldh18a1,Chadl,Cotl1, 1110001J03Rik,Scn5a,Fkbp5, Nrsn2,Sox2,6330403K07Rik, Sh3gl1,Sh3gl1,Ptges2,Whrn, Hdac5,Scrt2,B4galt3,Dner, Cnot8,Map3k4,Minos1,Flii, Plxnb1,Atn1,Hgs,Anp32e,Evl,Polr2c,Gfap,Tecr,Fam53c,Ptch1, Snrpa,Kctd17,Gtf2i,Acot7,Gnas,Hspa5,Pmm1,Mkl1,Dlst,Sbk1, Dctn1,Adcyap1r1,Rpl31,Tubb3, Jund,Ncs1,Abhd12,Rab1b, Polr2m,Ypel3,Slc25a22,Sh3bgrl3, Trim28,Sbf1,Trappc9,Rhobtb2, Rnf208,Sdha,Efhd2,Sgsm1*	*Actb,Ttll7,Rpl3,Meis2,Trim37,Chd4,Stmn2,Rtn4,Dtx3, Atp2b2,Hspa12a,Snn,Eif2s3x, Lin7a,Plxna4,Adrbk2,Tmod2, Atrn,Srcin1,Marf1,Sorbs2, Mfsd4,Mn1,Plekhg5,Rgs17, Fam98b,Zfp704,N28178, Slc30a3,Slc1a2,Rgs7bp,Lmo7, Dbpht2,Atxn3,Acap2,Atp6v0c,Kcnb1,Rock1,Cacna1d,Krt9, Nfat5,Erbb4,Plxdc1,Glt8d1, Myo9b,Rgs9,Ttc4,Coch,Sytl2, Cplx3,Gabpb2,Irgm2,Fndc1, Zbed6*
miR-764-3p	−1.275	*Zfp36,Tmem107, 2210013O21Rik,Akr7a5, 2310022A10Rik,Ube2s, Dpysl5,Ergic3,Slc41a1, Tmem179,Syn1,Prdx6,Ngef, Trim28*	*Usp7,Rtn4,Snn,Vldlr,Mllt4, Rufy3,Znf512b,Plekhg5, Nrg3,Ralgapa1,Gas7,Per3, Cxcl12,Slc13a4*

**Table 3:** KEGG pathway enrichment of DEGs for miRNAs and lncRNAs.

Pathways	miRNAs-DEGs			lncRNAs-DEGs		
	EF^1^	*P*-value^2^	DEmiRs^3^	EF	*P*-value	DElncRs^4^
Nicotine addiction				6.59	2.06E-06	522
Long-term potentiation				6.40	2.98E-12	869
Cocaine addiction	3.58	2.56E-02	3			
Amphetamine addiction	3.20	2.16E-03	7	4.61	6.47E-08	772
Glutamatergic synapse	2.68	4.44E-03	5	4.75	2.20E-10	980
Retrograde endocannabinoid signaling				4.61	6.47E-08	745
Endocrine and other factor-regulated calcium reabsorption				3.84	2.63E-04	431
Morphine addiction	2.61	1.22E-02	7	3.14	7.61E-04	543
GnRH signaling pathway	2.44	1.23E-02	4			
Long-term depression	2.34	4.43E-02	4	3.70	1.85E-04	449
Dopaminergic synapse	2.20	1.65E-02	5	4.35	2.88E-10	946
Calcium signaling pathway				3.39	1.04E-07	1083
GABAergic synapse				3.01	1.09E-03	561
Synaptic vesicle cycle				2.99	1.17E-03	494
Cholinergic synapse				2.98	4.43E-04	603
MAPK signaling pathway	2.07	2.40E-03	9	2.32	2.41E-05	1119
Neurotrophin signaling pathway				2.27	1.48E-03	692
Alzheimer’s disease				2.09	5.92E-03	603
Axon guidance				2.08	2.19E-03	861
Regulation of actin cytoskeleton				2.04	1.17E-04	1225
ErbB signaling pathway				3.46	2.94E-05	678
Insulin signaling pathway				2.89	1.41E-05	783
Phosphatidylinositol signaling system				2.67	1.86E-03	537
Wnt signaling pathway				2.34	1.07E-03	728
VEGF signaling pathway				2.19	2.26E-02	425
Adherens junction				2.76	9.02E-04	541
Focal adhesion				2.21	8.30E-05	1034
Gap junction				2.19	3.03E-02	377
Type II diabetes mellitus				3.67	3.85E-04	460
Ubiquitin mediated proteolysis				2.24	2.32E-03	661
Fatty acid elongation	9.31	4.07E-05	2			
Cysteine and methionine metabolism	3.74	1.10E-02	5			
Oxidative phosphorylation	3.06	4.83E-03	5			
Biosynthesis of secondary metabolites	2.24	9.44E-04	10			
Arginine and proline metabolism	3.41	8.57E-03	4			
Vibrio cholerae infection	3.03	3.11E-03	4	2.59	8.01E-03	459
mTOR signaling pathway	2.73	2.35E-02	3	3.63	4.22E-04	554
T cell receptor signaling pathway				2.27	7.06E-03	580
B cell receptor signaling pathway				2.20	1.62E-02	429
Protein export	9.09	2.05E-04	3			
Ribosome	3.15	1.40E-03	4			
Spliceosome	2.43	4.25E-03	3			

^1^Indicated enrichment factor.

^2^*P* values were calculated via hypergeometric tests and go with a correction.

^3^Indicated number of differentially expressed miRNAs^17^.

^4^Indicated number of differentially expressed lncRNAs^18^.
