# Supplementary material for: mRNA changes in nucleus accumbens related to methamphetamine addiction in mice
Source: Sci Rep. 2016 Nov 21;6:36993. doi: 10.1038/srep36993 (PMC5116666; doi:10.1038/srep36993)
Supplement: Supplementary Information [file srep36993-s1.pdf]

mRNA changes in nucleus accumbens related to methamphetamine addiction in mice

Li Zhu [1, 2], Jiaqi Li[1, 2], Nan Dong[1, 2], Fanglin Guan[1, 2], Yufeng Liu[3],Dongliang Ma[4,5], Eyleen L. K. Goh[4,5,6,7]& Teng Chen [1, 2, *]

[1]College of Forensic Medicine, Xi'an Jiaotong University Health Science Center, Xi'an, Shaanxi, 710061, PR China

[2]The Key Laboratory of Health Ministry for Forensic Science, Xi'an Jiaotong University, Shaanxi, PR China

[3]Beijing Genomics Institute, Shenzhen, 518083, PR China

[4]Neuroscience Academic Clinical Programme, Duke-NUS Medical School, Singapore169857

[5]Department of Research, National Neuroscience Institute, Singapore 308433

[6]Department of Physiology, Yong Loo Lin School of Medicine, National University of Singapore, Singapore 117597

[7]KK Research Center, KK Women's and Children's Hospital, Singapore229899

[*] Corresponding author: Teng Chen, College of Forensic Medicine, Xi'an Jiaotong University Health Science Center, Xi'an 710061, PR China

Tel/ Fax: +86 29 82657977; E-mail: chenteng@xjtu.edu.cn

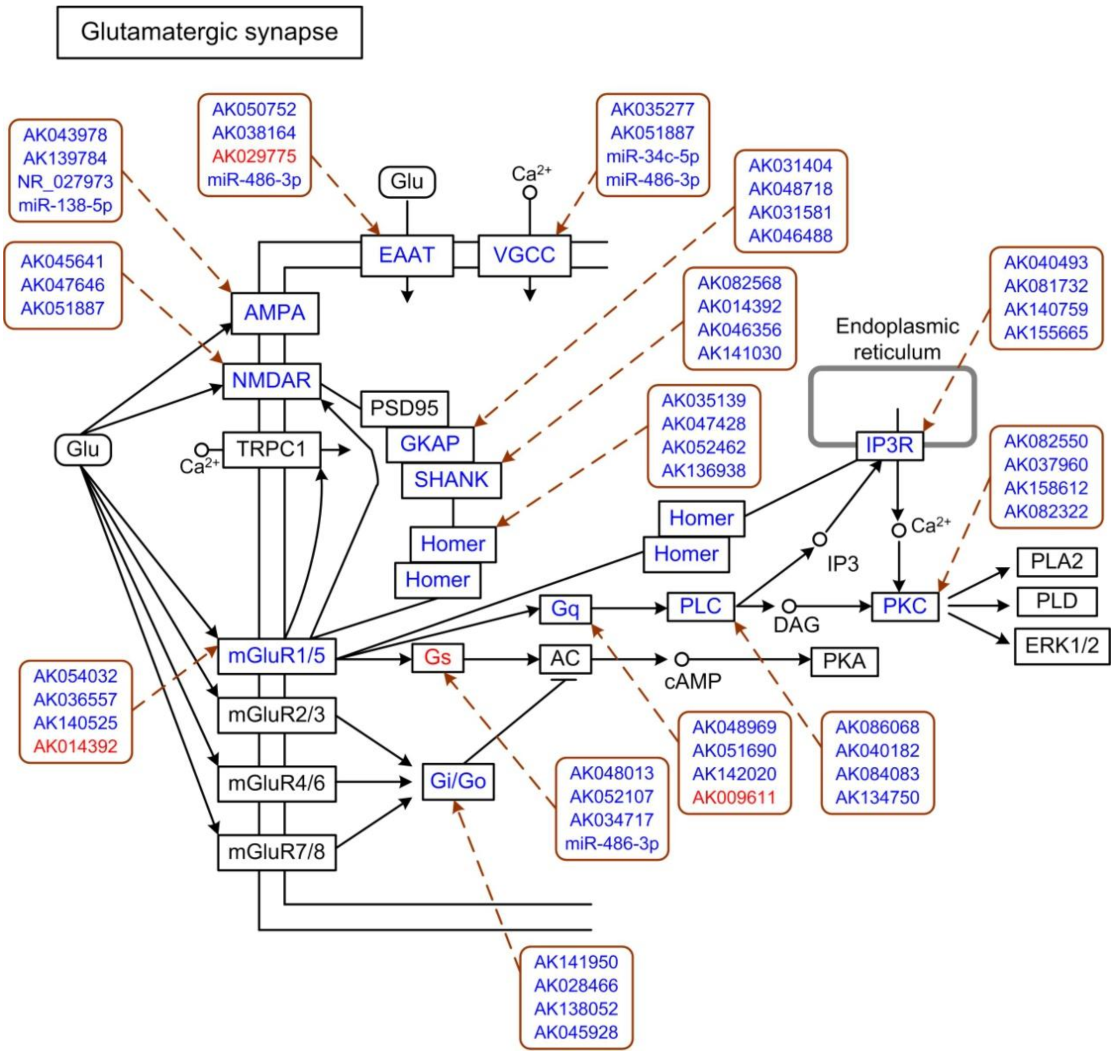

Supplementary Figure S1 Glutamatergic synapse pathway composed of DEGs and their corresponding miRNAs and lncRNAs.

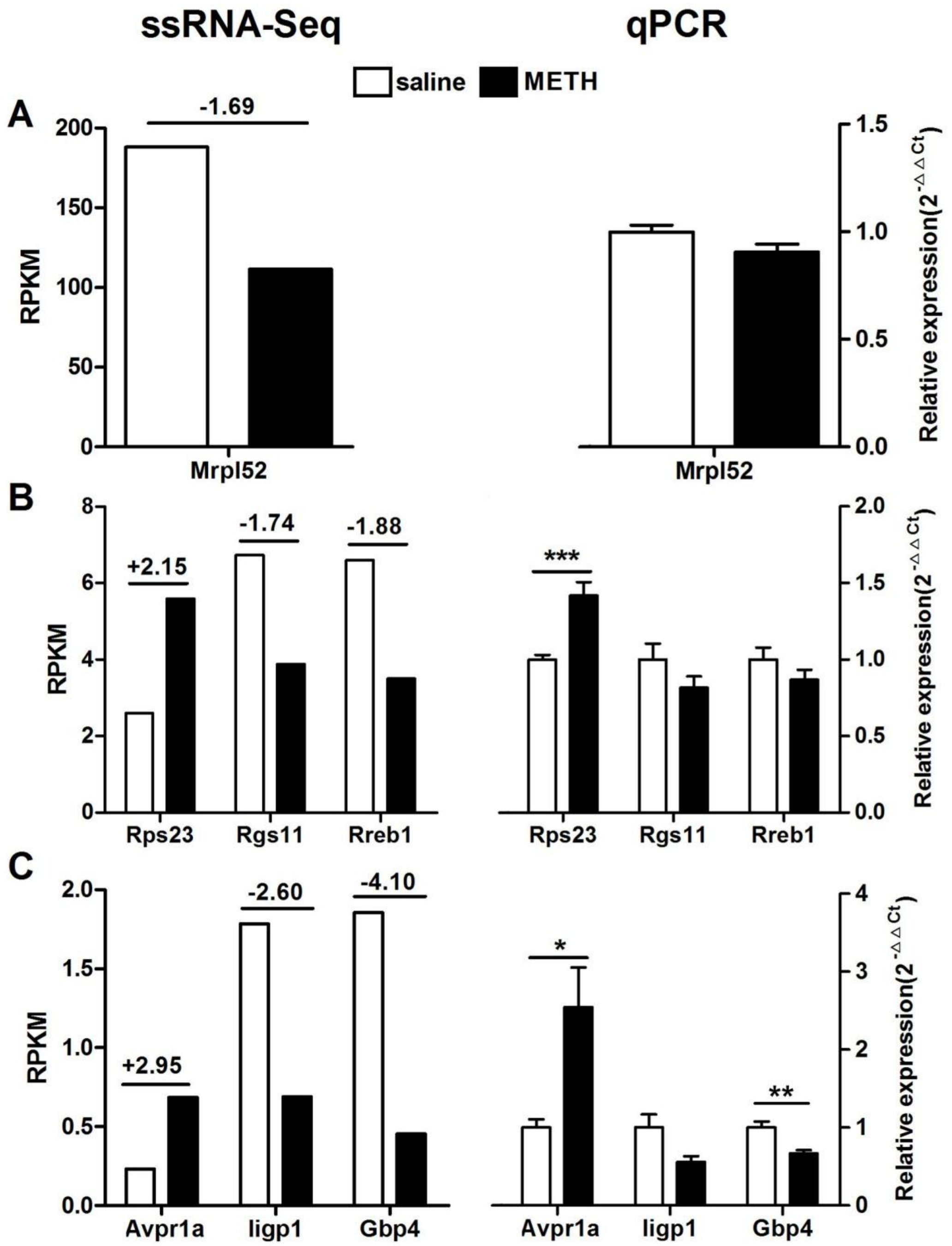

Supplementary Figure S2 Confirmatory qPCR

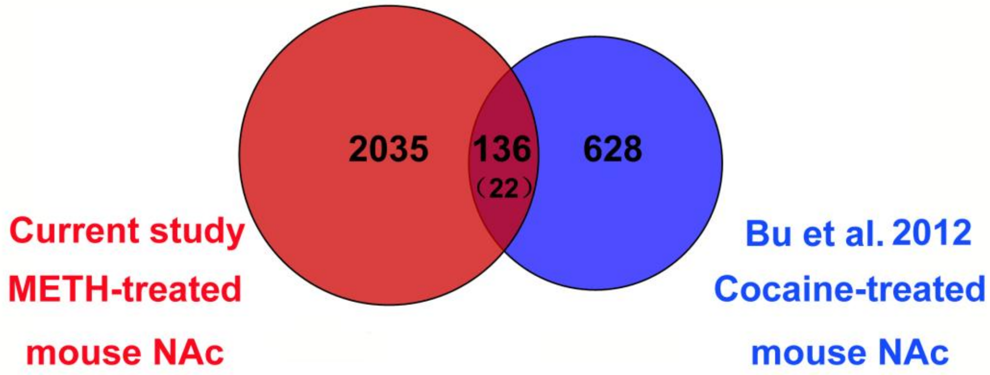

Gene	METH	Cocaine
Hist1h4h	↑	↓
Junb	↑	↓
Rarres2	↑	↓
Ntrk1	↑	↓
Lhx8	↑	↓
Chat	↑	↓
Igsf1	↑	↓
Slc18a3	↑	↓
Arhgap36	↑	↓
Cry1	↑	↓
Ramp3	↑	↓

Gene	METH	Cocaine
Neurod6	↓	↑
Pdzrn3	↓	↑
Rbm33	↓	↑
Cd74	↓	↑
Satb2	↓	↑
Tmsb15b2	↓	↑

Gene	METH	Cocaine
Gprin3	↓	↓
Ccdc57	↓	↓
Zmym6	↓	↓
Tyms	↓	↓
Ptprv	↓	↓

Supplementary Figure S3 136 DEGs in response to METH is involved in cocaine addiction. (A) Venn diagram showed the common set of 136 DEGs in NAc of mice following METH exposure (current study) and cocaine-treated mice[15]. (B) Directionality of expression changes in common set of 22 DEGs referred to in A.

Supplementary Table S2 qPCR primers

Gene	Forward primer (5'-3')	Reverse primer (5'-3')
Avpr1a	GGCGTTACTGGCTTCCTTGA	CCGAGTCATCCTTGGCGAAT
Junb	TCTACACCAACCTCAGCAGTTAC	TACGGTCTGCGGTTCCTCTT
Rps23	TGAAGTTCTGGTTGCTGGATT	GCCTTTCTTTCTTGCCTTTGTA
Ntrk1	CCAAGTCAGCGTCTCCTTCC	AGCACAGAGCCGTTGAACAA
Fos	GGTGAAGACCGTGTCAGGAG	CCGCTTGGAGTGTATCTGTCA
Mrpl52	AGACGAGTTGTACTGCTGAC	TGAGATCATGTTCCTTCTTCCT
Rgs11	ATTGACAGCAGGACGATGGA	TCAGACTTCAGGAACCTTGGAT
Rreb1	CTCCTCTTGACGCTGCTTCT	CTACTGTCTGGCTGGATGATGA
Iigp11	TTGCTGCTGACCTAGTGAATATC	CTCCACCTGATCCACCTCTATT
Gbp4	GCCATCATCGAGAGTTCCATC	GCTCCTTCTGCTTCTGTCTTAG
Ptprv	CTGTCTGTCTGGTGGAGGTAG	AGTGAGGCTAAGGCGGTAAG
Dicer1	GAATTGCTCGAGATGGAACCAGA	AGCTCCGGCCAACACCTTTA
Gapdh	TGTGTCCGTCGTGGATCTGA	TTGCTGTTGAAGTCGCAGGAG
